# Mercury Exposure Among Artisanal and Small-Scale Gold Miners in Four Regions in Uganda

**DOI:** 10.5696/2156-9614-10.26.200613

**Published:** 2020-05-28

**Authors:** Mercy Wendy Wanyana, Friday E. Agaba, Deogratias K. Sekimpi, Victoria N. Mukasa, Geoffrey N. Kamese, Nkonge Douglas, John C. Ssempebwa

**Affiliations:** 1 Uganda National Association of Community and Occupational Health, Kampala, Uganda; 2 Ministry of Gender Labour and Social Development, Kampala, Uganda; 3 Department of Disease Control and Environmental Health, School of Public Health, College of Health Sciences, Makerere University, Kampala, Uganda

**Keywords:** artisanal and small-scale gold miners, blood and urine mercury, mercury exposure mercury poisoning related symptoms

## Abstract

**Background.:**

Artisanal and small-scale gold mining is a human health concern, especially in low-income countries like Uganda due to the use of mercury (Hg) in the mining process.

**Objective.:**

The aim of the present study was to assess Hg exposure among artisanal and small-scale gold miners in Uganda through biologic monitoring parameters and Hg-related clinical manifestations.

**Methods.:**

A cross-sectional study was conducted from June to July 2018 among 183 miners from Ibanda (Western region), Mubende (Central region), Amudat (Karamoja region) and Busia (Eastern region) in Uganda. An interviewer-administered questionnaire and health assessment were used to collect socio-demographic, exposure and self-reported Hg poisoning symptoms. In addition, 41 urine, 41 blood and 26 environment samples were assessed. Descriptive statistics, Kruskal-Wallis test and Wilcoxon signed-rank test for comparison of Hg levels in urine and blood among miners were performed while logistic regression was used to assess associations between exposure and Hg poisoning-related symptoms.

**Results:**

The miners ranged from 15 to 65 years old and were primarily male (72.6%). The majority (73.3%) had worked directly with Hg for an average duration of 5.3 years. Symptoms associated with working directly with Hg included chest pain (odds ratio (OR)=9.0, confidence interval (CI)=3.3 to 24.6), numbness (OR=8.5, CI=2.1 to 34.4), back pain (OR=6.2, CI= 2.2 to 17.5), fatigue and stress (OR=5.4, 2.0 to CI=14.9), headache (OR=4.7, CI=1.9 to 11.3), dizziness (OR=3.8, CI=1.5 to 9.7) joint pain (OR=3.2, CI=1.3 to 8.3) and respiratory problems (3.2, 1.0 to 10.1). Statistically significant differences in Hg levels with p-values less than 0.05 were observed across district, gender and type of work. Mubende had the highest blood and urine levels (136 μg/l and 105.5 μg/l) in comparison with Busia (60 μg/l and 70.6 μg/l) and Ibanda (43 μg/l and 58 μg/l). Females (84.7 μg/l), panners (109 μg/l) and those with knowledge of occupational health and safety measures (95.6 μg/l) reported higher levels of Hg in urine. The average levels of Hg in water and soil samples were 23.79 μg/l and 0.21 μg/l, respectively.

**Conclusions.:**

Variation in Hg levels were attributed to varied duration of exposure across geographical sites. There was considerable exposure to Hg as indicated by both clinical manifestations and biologic parameters among miners in Uganda with Hg in urine exceeding the recommended thresholds.

**Participant Consent.:**

Obtained

**Ethics Approval.:**

Ethical approval was obtained from the Makerere University School of Health Science Institutional Review Board (reference number SHSREC REF 2018–2019) and Uganda National Council for Science and Technology (reference number SS 4577)

**Competing Interests.:**

The authors declare no competing financial interests.

## Introduction

Mercury (Hg) use in artisanal and small-scale gold mining (ASGM) is globally the largest source of occupational health exposure to Hg.^1^ In this type of mining, Hg is used to extract gold from its ore by forming an amalgam, which is then burnt, leaving the final gold product.^2,3^ These extraction processes expose artisanal and small-scale gold miners to Hg.

Although there are different forms of Hg with varying health effects, miners are mainly exposed to elemental Hg.^4,5^ Miners are exposed to its liquid form through skin contact during the panning process of forming the gold Hg amalgam and the vaporized form through inhalation during the burning process.^1,6^ Acute exposure to Hg through skin contact could lead to dermatitis.^7^ Similarly, acute exposure through inhalation could lead to erosive bronchitis and bronchiolitis with symptoms of sore throat, chest pain and shortness of breath.^7–9^ Other symptoms associated with acute exposure could include headaches, diarrhea, abdominal pain metallic taste, fever, numbness and general weakness.^8,10^ Chronic exposure could lead to neurological dysfunction with symptoms like fatigue, dizziness, weakness, tremors and shaking of hands.^9,11^ Erethism may also be observed with symptoms such as depression, irritability memory impairment and insomnia.^7,9^ Kidney problems such as proteinuria and nephrotic syndrome are associated with chronic exposure.^9,11^ Other conditions associated with chronic exposure may include gingivitis, eye irritation, joint pain stomatitis and excessive salivation.^9,11^ In some cases, miners could be exposed to methyl Hg as a result of consuming foods, including fish, from environments contaminated with Hg.^12^ Exposure to inorganic Hg may also occur through drinking water from Hg-polluted water sources.^13^ Exposure to elemental Hg can be monitored using urine samples, while both elemental and methyl Hg can be monitored using blood samples.^14^

With the rapid growth of the ASGM industry, especially in Uganda, Hg exposure in this sector is of major concern.^2,15^ By 2016, there were 150,000 people working directly as miners with 900,000 dependent on the trade, a number which is expected to grow.^15^ Although previous research has indicated Hg use among miners in Uganda, little is known about the level of exposures and whether the miners have experienced Hg poisoning-related symptoms.^15^ The present study sought to assess Hg exposure among miners in Uganda through biologic monitoring parameters and Hg-related clinical manifestations.

**Abbreviations**
ASGM: Artisanal and small-scale gold mining
OR: Odds ratios
OHS: Occupational health and safety
WHO: World Health Organization

## Methods

This was a cross-sectional study employing quantitative methods conducted between June and July 2018. The assessment included the use of the World Health Organization (WHO) standardized questionnaire for assessing Hg exposure, health assessment, human bio-monitoring and ecological monitoring.^16^

### Study area and population

**Figure 1:**
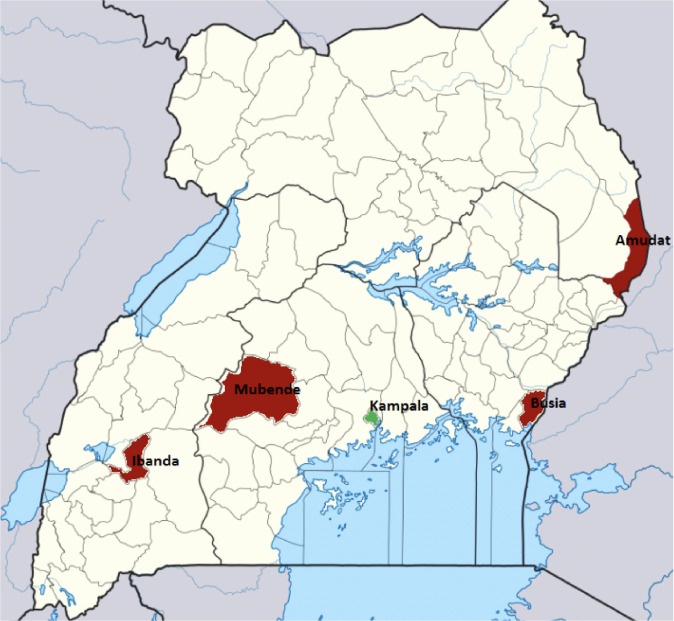
Map indicating selected mining districts in Uganda. Adapted from the Uganda Population and Housing Census 2014^20^

The present study was conducted in four districts with active artisanal and small-scale gold mines in the four regions where gold deposits are found: Ibanda (Western region), Mubende (Central region), Amudat (Karamoja region) and Busia (Eastern region) as shown in Figure 1. Some mining sites were located close to the shores of Lake Victoria. There are approximately 600, 2500, 3000 and 900 mines in Amudat, Busia, Mubende and Ibanda, respectively.^17^ However, these figures may not be accurate as the sector is largely illegal and disorganized with minimal documentation.^18^ These areas are generally rural with different types of geological formations including metasediment-hosted mesozonal mines in Mubende, intrusion-hosted mesozonal and sandstone-hosted mesozonal mines in Ibanda, mesozonal mines near the lake shores in Busia and Archean basement rocks and the upper amphibolite–lower granulite facies rocks with numerous hypozonal shear zone-controlled gold workings in Amudat.^19^ Study participants were selected from miners at two mining sites in each of the four mining districts in the four regions in Uganda. Selected mining sites included Kicuzi and Rukiri in Ibanda, Kitumbi and Lubali in Mubende, Mayeero and Tiira in Busia and Cheputakolo and Chepkarata in Amudat. Only miners who had worked for at least the past six months in the selected mining site were included in the study.

### Sampling plan

A sample of 160 was determined using the Leslie Kish formula assuming a prevalence of 5%, α=5%, and 10% non-response rate.^21^ Multi-stage sampling was used to select the final sample. Cluster sampling was used to randomly select one district within each region (cluster). Two mining sites were then randomly selected from each of the selected districts. The final sample was drawn from these mining sites using stratified sampling (strata included extractors (diggers, ore carriers, driers, stone crushers), processors (including panners) and burners/buyers).

### Health assessment

Health assessment was carried out by a registered clinical officer. This included a neurological examination, as well as evaluation of pre-existing conditions (for example malaria), tremors, sleep disturbances, fatigue, well-being and memory disturbances based on the WHO guidance on identifying populations at risk from Hg exposure.^16^ In addition, the clinical officer obtained self-reported symptoms experienced by participants in the past 6 months.

### Human biomonitoring

Human biomonitoring included blood and urine samples for total Hg determination. For biomarker data, 40 participants were randomly selected using a table of random numbers from the total 160 participants. A total of 41 urine and 41 blood samples were collected. Blood samples included 11 from Ibanda, 10 from Busia, 10 from Mubende and 10 from Amudat. Similarly, urine samples included 11 from Ibanda, 10 from Busia, 10 from Mubende and 10 from Amudat. However, due to challenges in transportation and logistics, Amudat samples were excluded from analysis. Sample collection, packaging, storage, and transportation procedures were based on the WHO/United Nations Environment Programme (UNEP) protocols for sample collection procedures for urine and blood.^16^ Five (5) mL of blood were drawn into an already labeled vacutainer and labeled with the participant's study number. The vacutainers were placed in a cool box. The same participant was given a urine container already labeled with her/his study number to collect about 20 mL of urine. The urine sample was again handled according to the recommended WHO standards for handling infectious samples. The urine container was placed in another cool box. As soon as 10 samples of blood and urine were obtained, the cool boxes were handled according to recommended standards and transported to the Directorate of Government Analytical Laboratory within a few hours according to the WHO recommended procedures.^16^

### Environmental monitoring

A total of 26 ecological samples were collected. This included water samples from drinking water sources and topsoil within a 20 m radius from the mining sites. Samples were stored and transported to the Directorate of Government Analytical Laboratory in Kampala based on an ecosystem sampling plan.

### Laboratory analysis

Laboratory analysis focused on the presence and levels of total Hg (mean-value Hg level). The Directorate of Government Analytical Laboratory carried out all blood, urine and the ecological sample analysis. Samples were prepared by digestion of sample with nitric, perchloric, and sulphuric-potassium permanganate solution. Testing for Hg was done using Shimadzu AAS-6300, the Hg vapor unit technique with a limit of detection of =<.0.001 was used. No reference materials were used.

### Data analysis

The data were analyzed using STATA version 12.0. Descriptive analysis was used to generate information on the use of Hg, average level of Hg in urine, blood and environmental samples. Categorical data were summarized using frequencies and percentages. Numerical data were summarized using means and SD for normally distributed data, and median and inter-quartile range for data that was not normally distributed. Chi-square tests were used to assess differences in self-reported symptoms across age group, gender and site location. Odds ratios (ORs) were used to analyze the relationship between Hg-related symptoms and Hg exposure. A logistic regression model was used to adjust for potential confounders. A backward-stepwise method was used to eliminate variables with p >0.05 in the process to adjust for potential confounders including neurological disorders, malaria, handling kerosene, smoking, alcohol use, pesticide use, use of whitening soap, hepatitis and tuberculosis.^16^ The findings were reported as ORs and confidence intervals (CI), and independent variables found to have p value of ≤0.0 and 95% CI, not including 1, were considered statistically significant in the final model. Non-parametric tests including Kruskal-Wallis test and Wilcoxon's signed rank test were used to compare Hg levels in urine and blood among different categories of miners.^22^

### Ethics

Ethical approval was obtained from the Makerere University School of Health Science Institutional Review Board (reference number SHSREC REF 2018–2019) and Uganda National Council for Science and Technology (reference number SS 4577). Informed consent was obtained from study participants and data anonymized. Biosafety measures of handling human samples were applied.

## Results

A total of 183 respondents participated in the study. Some participants who completed the questionnaire and were then selected for sample collection refused to have blood and urine samples taken. This led to collecting more questionnaire data from additional respondents in order to reach the target number of blood and urine samples. The analysis included all those who completed the questionnaire. Participants ranged between 15 to 65 years of age with most (50.3%) below 30 years. The majority were males (72.68%). The highest proportion (47.54%) had attained primary education as their highest level of education. The majority (68.3%) earned less than an equivalent of 540 US dollars annually. Details of the characteristics of the respondents are shown in Table 1.

**Figure 2:**
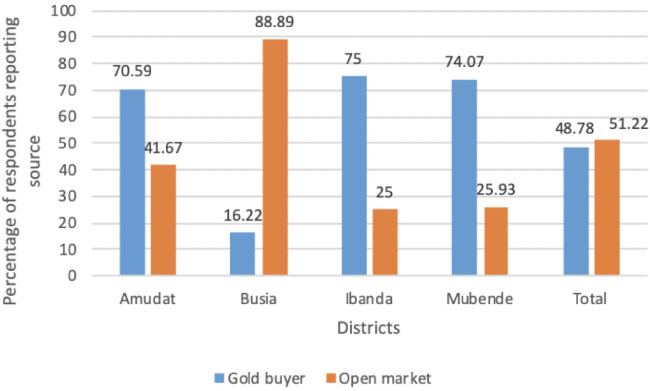
Sources of mercury per district

**Table 1:** Socio-Demographic Characteristics of Respondents

	**Amudat % (N)**	**Busia % (N)**	**Ibanda % (N)**	**Mubende % (N)**	**Total % (N)**
Gender					
Males	49.02 (25)	74.51 (38)	92.31 (36)	80.00 (32)	72.68(133)
Females	50.98 (26)	25.49(13)	7.69 (3)	20.00 (8)	27.32 (50)
Marital status					
Married or living together	84.31 (42)	80.39 (41)	76.92 (30)	76.19(32)	79.78 (146)
Divorced, separated, widowed	7.84 (4)	15.69 (8)	10.26 (4)	2.38(1)	9.29(17)
Never married					
	7.84 (4)	3.92 (2)	12.82 (5)	21.43 (9)	10.93 (20)
Highest level of education attained					
No education	21.57(11)	13.73 (7)	5.13 (2)	2.38(1)	11.48 (21)
Primary	50.98 (26)	37.25 (19)	56.41 (22)	47.62 (20)	47.54 (87)
Post primary education	27.45 (14)	49.02 (25)	38.46 (15)	50.00(21)	40.98 (75)
Religion					
Christian	86.27 (44)	92.16(47)	97.44 (38)	90.48 (38)	9.26(167)
Muslim	13.73 (7)	7.84 (4)	0	7.14(3)	7.65 (14)
Others	0	0	2.56(1)	2.38(1)	1.09 (2)
Annual income equivalent					
0–540 US dollars	78.43 (40)	80.39 (41)	64.19 (25)	45.24 (19)	68.31 (125)
Above 540 US dollars	21.57(11)	19.61 (10)	35.90 (14)	54.76 (23)	31.69 (58)
Age Group					
≤ 30 years	58.82 (30)	41.8(21)	38.46 (15)	61.90(25)	50.27 (92)
>30 years	41.18(21)	58.82 (30)	61.54(24)	38.10(16)	49.73 (91)

### Use of mercury at mining site

The use of Hg was reported in all mining sites. Overall, use was reported by approximately 81% of the miners. The highest usage was in the Amudat and Buisa districts (100%) compared to Mubende (73.7%) and Ibanda (41.2%). The most prevalent source of Hg was the open markets (51.2%) within the mines. Open markets included shops selling other items located near the mines. Gold buyers also supplied miners with Hg for processing gold.

### Occupational exposure to mercury

Occupational exposure was assessed based on the WHO questionnaire. Participants were asked whether they were directly exposed to Hg through working directly with Hg. The majority (73.3%) of miners reported they had at some point worked directly with Hg, 88.2% in Busia ; 86.3% in Amudat; 72.50% in Mubende; and 36.84% in Ibanda. The average duration (mean) years of exposure was 5.35 years (95% CI: 4.20 to 6.49), with Busia having the longest duration of exposure, 9.97 years (7.51 to 12.44), compared to other districts. Although the majority (75.8%) of respondents reported using personal protective equipment, this was primarily in the form of wearing gumboots. Details of occupational exposure are presented in Table 2.

**Table 2:** Occupational Mercury Exposure

	**Amudat % (N)**	**Busia % (N)**	**Ibanda % (N)**	**Mubende % (N)**	**Total % (N)**
Ever worked directly with mercury					
Yes	86.3 (44)	88.2 (45)	36.8(14)	72.5 (29)	73.3 (134)
No	13.7 (7)	11.8 (6)	63.2 (24)	27.5(11)	26.7 (49)
Duration in years working directly with mercury					
Mean, SD (95% CI)	3.59,(2.511 to 4.68)	9.97,(7.51 to 12.44)	1.02, (0.38 to 1.65)	2.45, (1.80 to 3.10)	5.35, (4.20 to 6.49)
Ever worked burning amalgam in open pans or melting gold in inadequate fume hoods					
Yes	39.6(19)	61.2 (30)	25.7 (9)	43.6(17)	43.9 (75)
No	60.4 (29)	38.8(19)	74.3 (26)	56.4 (22)	56.1 (96)
Mean, SD (95% CI)	3.23, (1.77 to 4.70)	8.36, (5.71 to 11.02)	2.06, (0.58 to 3.55)	2.20,(1.41 to 2.99)	4.99, (3.68 to 6.31)
Ever stored mercury at home					
Never	43.1 (22)	18.0 (9)	70.3 (26)	53.9 (21)	44.1 (81)
At work	11.8 (6)	16.0 (8)	18.9 (7)	20.5 (8)	16.4 (30)
At home	45.1 (23)	66.0 (33)	10.8 (4)	25.6(10)	39.6 (72)
Average distance (in km) of processing site to miner’s residence					
Less than 1 km	45.1 (23)	49.0 (25)	71.8 (28)	88.1 (37)	77.1 (141)
1–3 km	33.3 (17)	29.4 (15)	18.0 (7)	11.9 (5)	14.6 (27)
3.1–5 km	11.8(6)	13.7 (7)	10.3 (4)	0	6.0(11)
More than 5 km	9.8 (5)	7.8 (4)	0	0	2.19(4)
Use personal protective equipment					
Yes	4.1 (2)	14.0 (7)	18.0 (7)	32.5(13)	24.2 (44)
No	95.9 (47)	86.0 (43)	82.0 (32)	67.5 (27)	75.8(139)

### Environmental exposure

Ecological sample analysis indicated Ibanda had the highest (31.8 μg/l) mean Hg levels in water as shown in Table 3. Mubende had the highest (0.28 μg/l) soil mean Hg levels. The Hg levels were above the maximum acceptable level of Hg (6 μg/l) based on WHO guidelines for drinking water quality.^23^

**Table 3:** Total Mercury Levels in Soil and Water Samples

	**Mean Hg (μg/l)**	

**District**	**Water**	**Soil**
Ibanda	31.8	0.26
Busia	28.33	0.08
Mubende	11.25	0.28

### Health effects associated with exposure to mercury

A clinical officer asked respondents whether they had experienced previously documented Hg-related symptoms in the last 6 months. When all significant variables from the bivariate analysis were transferred to a multivariable logistic regression model and the effect of potential confounders (neurological disorders, malaria, handling kerosene, smoking, alcohol use, pesticide use, use of whitening soap, hepatitis and tuberculosis) were controlled for, study variables maintained significance *(Table 4).* Statistically significant associations were established with p ≤0.05 and a 95% CI, not including zero. Symptoms such as swelling of feet, a metallic taste in the mouth, painful feet (perceived as holes in feet by miners), excessive salivating, loss of appetite and loss of hair, although reported, were not associated with using Hg.

**Table 4:** Crude and Adjusted Associations for Symptoms Associated with Mercury Exposure

**Symptoms**	**Exposed (yes) % (N)**	**Crude OR**	**95% CI**	**OR (adjusted of known confounding factors)**	**95% CI**
Shaking of hands and head^*^	88.57 (31)	7.75	2.74 to 21.96	24.09	1.71 to 338.74
Eye problems^*^	90.20 (46)	9.20	3.66 to 23.15	10.97	1.97 to 62.48
Chest pain^*^	88.89 (72)	8.00	4.00 to 16.00	9.02	3.31 to 24.60
Numbness^*^	88.71 (55)	7.86	7.86 to 3.58	8.51	2.11 to 34.36
Back pain^*^	85.88 (73)	6.08	3.30 to 11.20	6.21	2.20 to 17.50
Fatigue and stress^*^	86.75 (72)	6.54	3.47 to 12.34	5.38	1.94 to 14.88
Headache^*^	86.42 (70)	6.36	3.36 to 12.01	4.67	1.93 to 11.28
Dizziness^*^	85.71 (54)	6.00	2.94 to 12.15	3.84	1.52 to 9.74
Joint pain^*^	85.96 (49)	6.12	2.90 to 12.93	3.23	1.26 to 8.33
Respiratory problems^*^	87.18(34)	6.8	2.65 to 17.38	3.18	1.01 to 10.12

^*^Statistically significant association with p value less than 0.05 and 95% CI not including zero.

Adjusted OR obtained from a logistic regression model following adjustment of potential confounders (including neurological disorders, malaria, handling kerosene, smoking, alcohol use, pesticide use, use of whitening soap, hepatitis and tuberculosis).

Furthermore, statistically significant differences in self-reported symptoms were observed across gender and geographical location as detailed in Supplemental Material. Ibanda had the lowest proportion of respondents reporting experiencing these symptoms in comparison with the other districts. More females reported experiencing diarrhea, stomachache, swelling of legs, poor memory and respiratory problems compared to males, while more males reported injuries. These differences were statistically significant with p-values less than 0.05 as indicated in Supplemental Material. No statistically significant differences were indicated across age.

### Mercury levels in blood and urine

Mercury levels in blood ranged between 26.3 μg/l to 205 μg/l with a median blood level of 67.5 μg/l, while Hg levels in urine ranged between 37.5 μg/l to 296 μg/l with a median urine level of 70.8 μg/l.

Mubende district had the highest median blood levels of Hg (136 μg/l) relative to Busia (60 μg/l) and Ibanda (43 μg/l). Based on the Kruskal-Wallis equality-of-populations rank test, these differences were statistically significant (chi square value χ^2^=15.147, df=2 and p-value=0.0005). Similarly, Mubende district had the highest median urine levels of Hg (105.5 μg/l) relative to Busia (70.6 μg/l) and Ibanda (58 μg/l). Using the Kruskal-Wallis test, there was a statistically significant difference in the median Hg urine levels across districts (chi square value χ^2^=11.664, df=2 and p-value=0.0029).

With regard to differences in Hg levels between socio-economic and occupational mining categories, statistically significant differences were obtained between Hg levels in urine across gender, type of work and knowledge of occupational health and safety (OHS) practices. Males had a lower median level of Hg in urine (65.4 μg/l) compared to females (84.7 μg/l). There was a difference of 69.6 (95% C1: 62.8 to 73, p-value of 0.0476, which is less than 0.05), and statistically significant. Similarly, panners had the highest Hg levels in urine (109 μg/l) compared to burners (90.6 μg/l) and extractors (62.9 μg/l). This observed difference was statistically significant with a chi square value χ^2^= 9.595, df=2 and p-value=0.0083, which was less than 0.05.

Those with knowledge of OHS practices had higher median levels of Hg in blood and urine (119.5 μg/l and 95.6 μg/l, respectively) than those without knowledge of OHS practices (52.25 μg/l and 65.4 μg/l, respectively). The median difference in blood Hg levels for those with and without knowledge of OHS practices was −66 (95% CI: −102.4 to −51.5) with a p-value of 0.0129, which is less than 0.05. The median difference in urine Hg levels for those with and without knowledge of OHS practices was −69 (95% CI: −72 to −61.8) with a p-value of 0.0280, which is less than 0.05. This indicates statistically significant differences in both Hg blood and urine levels between those with knowledge of OHS practices and those without knowledge.

## Discussion

In Uganda, Hg use among miners has persisted, despite known negative health impacts.^24^ The present study sought to assess Hg exposure among miners through biologic monitoring parameters and Hg-related clinical manifestations in order to provide information needed for designing exposure prevention interventions.

Similar to findings in studies conducted in Ghana and Burkina Faso, miners in this study were characterized by low levels of education and low incomes.^23–25^ The high levels of miners with less than secondary level education (either no education or a primary level of education—59%) in this study are comparable to those documented in Ghana (70%) and Burkina Faso (75.5%).^25,27^ These characteristics may increase the likelihood of exposure as a result of limited awareness of the dangers of Hg use and limited available protective measures and inability to afford personal protective equipment.^5,28^ Nearly a third (27%) of the miners were female. Female active involvement in ASGM activities is likely to increase exposure of Hg to children living in these communities.^29,30^ Women in Africa work with their children in mining sites, thus exposing their children to Hg.^31^

This study demonstrates considerable occupational exposure to Hg through self-reported use (73.3%) and biologic monitoring parameters. Detection of Hg in blood indicates current exposure, while detection in urine indicates exposure that occurred sometime in the past.^16^ Average levels of Hg in blood and urine (67.5 μg/l and 70.8 μg/l, respectively) among miners are relatively higher than those obtained in Tanzania (14.6 μg/l and 46.3 μg/l, respectively) among miners with relatively similar education levels.^32^ Mercury levels among all miners in the Mubende, Busia and Ibanda districts were above the WHO's “Human Bio-Monitoring II” threshold levels of 15 μg/l for blood and 25 μg/l for urine, respectively.^16^ The proportions of miners with blood and urine levels above the recommended levels in this study were higher in comparison with previous studies in Burkina Faso, South Africa and Ghana.^25,33,34^ Fifty percent (50%) of miners in South Africa and 5%–46.7% in Ghana had Hg urine levels above the recommended levels.^20,33,33^ However, these differences are possibly attributed to differences in comparison guidelines. Whereas this study used a recommend level of 25 μg/l for urine, other studies used 50 μg/l.^21^ Furthermore, observed variation in Hg levels across the three regions could be attributed to the varied durations of exposure. Districts reporting higher levels of Hg in blood, such as the Mubende and Busia districts (2 years and 10 years, respectively), had longer durations of exposure in comparison to the Ibanda district (1 year). A high proportion (88%) of miners residing very close (less than 1 km) to mines could have further increased exposure among miners in the Mubende.^2^

Unlike a previous study in Ghana indicating higher levels of Hg in urine among males (1.38 μg/l) in comparison to females (0.53 μg/l), the present study indicated higher levels among females.^36^ These biologic parameter and clinical manifestation differences by gender could be attributed to different work-related gender roles in the various settings leading to variations in exposure. Gold mining activities such as panning are predominantly female roles in some contexts, like in the present study.^37^ This exposes them to Hg as it involves the use of metallic Hg to form the amalgam. In this study, panners had significantly higher levels of Hg compared to the rest of the occupational groups as seen in Table 5. These findings differ from a systematic review of studies conducted in the Philippines, Mongolia, Tanzania, Zimbabwe and Indonesia which indicated burners had higher levels of Hg in urine.^38,39^ Those with self-reported knowledge of OHS practices had higher levels of Hg in blood and urine compared to those without. This could be attributed to longer experience in ASGM and therefore perceiving oneself as more knowledgeable.^27^ Longer work duration in artisanal gold mining has been associated with longer duration of exposure and consequently greater accumulation of Hg in the body.^5,40^ Furthermore, it has been shown that knowledge alone may not always translate into safer occupational health practices.^41^ In addition to occupational exposure, miners living in the study communities may also experience environmental exposure as water samples collected in the area had levels of Hg above the recommended levels. Levels of Hg in this study are higher than those obtained from streams, rivers and boreholes in ASGM areas in Ghana that ranged from < 1 to 4 μg/l.^42^

**Table 5:** Differences in Blood and Mercury Levels by Miner Demographics

	**Blood**					**Urine**				

	**Median Hg level (μg/l)**	**Median difference Hg level (μg/l) (CI)**	**Rank sum**	**Z-score**	**p-value**	**Median Hg level (μg/l)**	**Median difference Hg level (μg/l) (CI)**	**Rank sum**	**Z-score**	**p-value**

**Mann Whitney U test**										

Gender										
Male	54.4	−66 (−103.4 to −51.5)	327	−1.447	0.1479	65.4	69.6 (62.8 to 73)	350.5	−1.981	0.0476^*^
Female	104.4		138			84.7		177.5		
Age group										
≤30 years	72.5	66 (51.1 to 102.4)	236.5	−0.478	0.6325	78.8	69 (62.8 to 72)	280.5	1.304	0.1924
>30 years	53.75		228.5			63.8		247.5		
Use of PPE										
Yes	119.5	−66 (−102.4 to −51)	158	454.46		79	−66 (−72 to 61.8)	133	0.044	0.9653
No	52.8		307		0.1107	69.7		395		
Knowledge on OHS										
Yes	119.5	−66 (−102.4 to −51.1)	177	2.486	0.0129^*^	95.6	−69 (−72 to −61.8)	219	2.197	0.0280^*^
No	52.25		288			65.4		309		

**Kruskal – Wallis test**										

	**Median Hg level (μg/l)**		**χ^2^**	**df**	**p-value**	**Median Hg level (μg/l)**		**χ^2^**	**df**	**p-value**

Education										
No education	65.6					68.6				
	52		4.541	4	0.3378	62.9		2.659	4	0.6164
Primary	61.25					75.4				
‘O’ level	120					53				
‘A’ level	128					96.8				
Tertiary										
Type of work										
Extractor	52.5		4.637	2	0.0987	62.9		9.595	2	0.0083^*^
Panner	172.5					109				
Burner	69.4					90.6				

Abbreviations: PPK, personal protective equipment, ‘O’ level, four years of secondary education; ‘ A ’ level, six years of secondary education

In the present study, miners who, at some point, had worked directly with Hg were more likely to experience some of the symptoms that have been suggested to be clinical presentations of Hg intoxication.^9^ These included shaking of the hands and head, eye problems, chest pain, numbness, back pain, fatigue, headaches, dizziness, respiratory problems and joint pain. However, like previous studies conducted in Tanzania and Ghana, there were no statistically significant differences for other known symptoms such as a metallic taste, excessive salivating, loss of appetite and loss of hair between those who had, at some point, worked with Hg and those who had not.^25,43^

### Limitations

There are some limitations to these findings. Firstly, due to the cross-sectional design there are limitations in establishing a causal relationship between Hg symptoms and Hg exposure.^44^ Second, wide CIs obtained with ORs may lower the precision of the obtained estimates.^45^ Third, the small sample sizes used in the analysis of Hg levels in blood and urine may affect the generalizations of the results.^46^ Fourth, no reference materials were used in the analysis of the samples, therefore the traceability of results could not be determined.^47^ Finally, self-reported data on symptoms may be susceptible to recall bias.^48^

## Conclusions

In conclusion, the findings of the present study provide evidence of high levels of Hg exposure among artisanal and small-scale miners in Uganda. These findings will be helpful in guiding future interventions aimed at preventing exposure to Hg in the ASGM sector and mining communities. Future studies should examine causal factors attributed to exposure among miners in order to design more effective interventions.
