# Supplementary material for: Mercury Exposure Among Artisanal and Small-Scale Gold Miners in Four Regions in Uganda
Source: J Health Pollut. 2020 May 28;10(26):200613. doi: 10.5696/2156-9614-10.26.200613 (PMC7269329; doi:10.5696/2156-9614-10.26.200613)
Supplement: Supplementary file 1 [file Wanyana_Supplemental.docx]

| Supplemental Material  Self-Reported Symptoms | | | | | | | | |
| --- | --- | --- | --- | --- | --- | --- | --- | --- |
| Symptom | **Gender** | | | **Regions** | | | | |
|  | **Males** | **Females** | **Chi-Square (p-value)** | **Mubende** | **Busia** | **Ibanda** | **Amudat** | **Chi-square (p-value)** |
| Injuries | 74.7% (53) | 25.3% (18) | 0.13 (0.716) | 57.1% (24) | 25.49% (13) | 25.5% (10) | 47.1% (24) | 14.06 (0.003)* |
| Headache | 65.4% (53) | 34.6% (28) | 4.25 (0.039)* | 38.1% (16) | 60% (26) | 23.1% (9) | 58.82% (30) | 13.06 (0.005)* |
| Swelling of legs | 7.5% (10) | 22.0% (11) | 7.5 (0.006)* | 11.5% (21) | 3.9% (2) | 2.6% (1) | 27.5% (14) | 18.88 (0.000)* |
| Numbness | 32.3% (43) | 40% (20) | 0.94 (0.331) | 21.4% (9) | 60% (26) | 30.7% (12) | 31.4% (16) | 9.78 (0.021)* |
| Stomachache | 19.6% (26) | 36% (18) | 5.38 (0.020)* | 19.1% (8) | 19.6% (10) | 10.3% (4) | 43.1% (22) | 15.36 (0.002)* |
| Shaking of hands | 18.8% (25) | 20.0% (10) | 0.034 (0.854) | 11.9% (5) | 19.6% (10) | 10.3% (4) | 31.4% (16) | 8.35 (0.039)* |
| Poor memory | 19.6% (26) | 34% (17) | 4.22 (0.040* | 14.3% (6) | 25.5% (13) | 23.1% (9) | 29.41% (15) | 3.09 (0.378) |
| Psychiatric disturbance | 6% (8) | 18% (9) | 6.19 (0.013) | 4.8% (2) | 2% (1) | 0 | 27.5% (14) | 28.23 (0.000)* |
| Painful feet | 10.5% (14) | 20% (10) | 2.86 (0.091) | 9.5% (4) | 5.9% (3) | 7.7% (3) | 9.5% (4) | 13.02 (0.005)* |
| Respiratory problems | 18.1% (24) | 32% (16) | 4.14 (0.042)* | 14.3% (6) | 9.8% (5) | 12.8% (5) | 47.1% (24) | 26.58 (0.000)* |
| Eye problems | 24.1% (32) | 38% (19) | 3.5 (0.061) | 19.1% (8) | 41.2% (21) | 15.4% (6) | 31.4% (16) | 9.45 (0.024)* |
| Joint pain | 28.6% (38) | 38% (19) | 1.50 (0.220) | 28.6% (12) | 33.3% (17) | 18% (7) | 41.2% (21) | 5.8 (0.122) |
| Diarrhea | 9.8% (13) | 24% (12) | 6.23 (0.013)* | 11.9% (5) | 7.8% (4) | 0 | 31.4% (16) | 21.3 (0.000)* |
| Chest pain | 42.9% (57) | 50% (25) | 0.74(0.387) | 33.3% (14) | 51% (26) | 30.8% (12) | 58.8% (30) | 10.18 (0.017)* |
| Back pain | 45.11% (60) | 52% (26) | 0.69(0.405 | 42.9% (18) | 60.8% (31) | 30.8% (12) | 49% (25) | 8.38 (0.039)* |
| General malaise | 22.6% (30) | 34% (17) | 2.49(0.114) | 26.2% (11) | 23.5% (12) | 7.7% (3) | 41.18% (21) | 13.15 (0.004)* |
| Dizziness | 31.6% (42) | 42% (21) | 1.74(0.186) | 19.1% (8) | 43.1% (22) | 20.5% (8) | 49% (25) | 14.27 (0.003)* |
| Fatigue and stress | 46.6% (62) | 44% (22) | 0.01(0.752) | 52.4% (22) | 45.1% (23) | 28.2% (11) | 54.9% (28) | 7.30 (0.063)* |
| *Statistically significant difference with p-value less than 0.05. | | | | | | | | |
|  | | | | | | | | |
